# Non-invasive diffuse optical neuromonitoring during cardiopulmonary resuscitation predicts return of spontaneous circulation

**DOI:** 10.1038/s41598-021-83270-5

**Published:** 2021-02-15

**Authors:** Tiffany S. Ko, Constantine D. Mavroudis, Ryan W. Morgan, Wesley B. Baker, Alexandra M. Marquez, Timothy W. Boorady, Mahima Devarajan, Yuxi Lin, Anna L. Roberts, William P. Landis, Kobina Mensah-Brown, Vinay M. Nadkarni, Robert A. Berg, Robert M. Sutton, Arjun G. Yodh, Daniel J. Licht, Wensheng Guo, Todd J. Kilbaugh

**Affiliations:** 1 Division of Neurology, Children’s Hospital of Philadelphia, 3401 Civic Center Blvd, Philadelphia, PA 19104 USA; 2 Division of Cardiovascular Surgery, Department of Surgery, Hospital of the University of Pennsylvania, 3400 Spruce Street, Philadelphia, PA 19104 USA; 3 Department of Anesthesiology and Critical Care Medicine, Children’s Hospital of Philadelphia, 3401 Civic Center Blvd, Philadelphia, PA 19104 USA; 4 Department of Physics and Astronomy, University of Pennsylvania, 209 South 33rd Street, Philadelphia, PA 19104 USA; 5 Department of Biostatistics and Epidemiology, University of Pennsylvania, 423 Guardian Dr, Philadelphia, PA 19104 USA

**Keywords:** Biomedical engineering, Translational research, Predictive markers, Preclinical research, Paediatric research

## Abstract

Neurologic injury is a leading cause of morbidity and mortality following pediatric cardiac arrest. In this study, we assess the feasibility of quantitative, non-invasive, frequency-domain diffuse optical spectroscopy (FD-DOS) neuromonitoring during cardiopulmonary resuscitation (CPR), and its predictive utility for return of spontaneous circulation (ROSC) in an established pediatric swine model of cardiac arrest. Cerebral tissue optical properties, oxy- and deoxy-hemoglobin concentration ([HbO_2_], [Hb]), oxygen saturation (StO_2_) and total hemoglobin concentration (THC) were measured by a FD-DOS probe placed on the forehead in 1-month-old swine (8–11 kg; n = 52) during seven minutes of asphyxiation followed by twenty minutes of CPR. ROSC prediction and time-dependent performance of prediction throughout early CPR (< 10 min), were assessed by the weighted Youden index (J_w_, w = 0.1) with tenfold cross-validation. FD-DOS CPR data was successfully acquired in 48/52 animals; 37/48 achieved ROSC. Changes in scattering coefficient (785 nm), [HbO_2_], StO_2_ and THC from baseline were significantly different in ROSC versus No-ROSC subjects (*p* < 0.01) after 10 min of CPR. Change in [HbO_2_] of + 1.3 µmol/L from 1-min of CPR achieved the highest weighted Youden index (0.96) for ROSC prediction. We demonstrate feasibility of quantitative, non-invasive FD-DOS neuromonitoring, and stable, specific, *early* ROSC prediction from the third minute of CPR.

## Introduction

Physiology-directed cardiopulmonary resuscitation (CPR) strategies are at the forefront of research to improve patient outcomes following pediatric in-hospital cardiac arrest (pIHCA)^1–3^. Current American Heart Association pediatric guidelines for high-quality CPR recommend performing chest compressions to a depth of at least one-third of the anterior–posterior diameter of the chest^4^. Hemodynamic-directed CPR (HD-CPR) is an emerging CPR strategy where physiologic-directed measurements of the patient, instead of solely depth and rate from the rescuer, are used to guide CPR^5^. HD-CPR has been shown to improve survival and neurological outcomes compared to depth-directed guideline CPR in pre-clinical models of cardiac arrest^6–11^. However, the need for invasive, indwelling vascular access to continuously transduce blood pressure during CPR precludes broad translation of this patient-centric (*i.e.*, versus rescuer-centric) strategy. Successful resuscitation is typically defined as the return of spontaneous circulation (ROSC); yet, among patients who achieve ROSC, neurologic injury is a leading cause of morbidity and mortality^12,13^. To broaden applicability and bridge the gap between resuscitation and neurologically favorable survival, it is imperative to incorporate novel, non-invasive physiologic feedback from the brain in the next generation of high-quality CPR strategies.

Non-invasive cerebral near-infrared spectroscopy (NIRS) has provided mixed evidence for addressing these challenges in adult populations^14^. Cerebral NIRS-measurements of regional cerebral tissue oxygen saturation (rSO_2_) during CPR have been associated with outcomes^14–16^. However, variability in measured values between different clinical NIRS devices is widely reported and suggests the value of more advanced optical approaches which ameliorate sources of variability stemming from discrepant analysis algorithms and physiologic assumptions^14,17^. Currently, all FDA-approved clinical NIRS devices employ continuous-wave (CW) light sources to measure rSO_2_^14^. Due to the limited information gathered, CW-NIRS devices cannot uniquely separate the effects of tissue optical scattering from absorption on measured CW intensities. This necessitates use of population-derived assumptions about the optical scattering coefficient (µ_s_′) which limits the accuracy of absorption spectroscopy-based rSO_2_ values during profound physiologic changes that can impact µ_s_′. Pediatric-specific adjustments of these assumptions, and inherent differences in arrest etiologies, limit the translation of NIRS findings in adult populations to pediatric populations where there is a paucity of data on the use of cerebral NIRS during CPR^18–20^. Further study and optimization of more advanced optical approaches which address these assumptions during CPR in pediatric populations is urgently needed.

In this contribution, we apply advanced non-invasive frequency-domain diffuse optical spectroscopy (FD-DOS) in an established pediatric model of in-hospital cardiac arrest^6–10,21^ to provide accurate, subject-specific *absolute* cerebral hemodynamics including oxy- and deoxy-hemoglobin concentrations ([HbO_2_],[Hb], respectively), total hemoglobin concentration (THC), and cerebral tissue oxygen saturation (StO_2_) via absolute and concurrent quantification of both tissue scattering and absorption properties using radio-frequency amplitude-modulated laser sources^22^. We demonstrate the feasibility of FD-DOS during CPR and assess the association and the predictive utility of intra-arrest *cerebral* hemodynamic measurements with and for ROSC. Using cross-validation and time-dependent analyses, we determine decision thresholds with consistent predictive performance throughout the first 10 min of CPR.

## Methods

Non-invasive FD-DOS measurements of cerebral hemodynamics were conducted in one-month-old (8–11 kg; n = 52) female swine (*sus scrofa domesticus*) during asphyxia and CPR. The measured subjects comprised a convenience sample derived from three separate randomized controlled trials in which animals were randomized to (a) depth-guided CPR versus hemodynamic-directed CPR^6^, (b) depth-guided CPR with versus without inhaled nitric oxide, or (c) hemodynamic-directed CPR using 100% versus 21% fraction of inspired oxygen (FiO_2_)^21^. Hemodynamic-directed CPR is a previously described strategy^6,7,11^ in which resuscitation therapies are titrated according to invasively measured blood pressures, as detailed below. FD-DOS measurements were not a primary outcome measure and were conducted based on personnel and equipment availability, thus monitoring was only performed observationally on a subset of these subjects. Detailed protocols of animal preparation, anesthetic maintenance, physiologic monitoring, and CPR parameters have been previously described in addition to considerations underlying the fidelity of this pre-clinical pediatric model of asphyxia-associated cardiac arrest^6,7^. All procedures were approved by the CHOP Institutional Animal Care and Use Committee, performed in strict accordance with the NIH Guide for the Care and Use of Laboratory Animals, and reported according to the ARRIVE guidelines (https://www.nc3rs.org.uk/arrive-guidelines).

### Frequency-domain diffuse optical spectroscopy (FD-DOS)

Continuous FD-DOS measurements of optical absorption and scattering properties (µ_a_ and µ_s_′, respectively) at wavelengths λ = 690, 725, 785, and 830 nm were acquired at 10 Hz using a customized commercial instrument (Imagent, ISS Inc., Champaign, IL, USA) and a corresponding optical probe secured onto the forehead. The instrumentation, analysis, and data quality criteria for these measurements have been previously described^23^. To characterize temporal changes in the relationship between wavelength and optical scattering, for each timepoint, the measured µ_s_′ across wavelengths $\lambda$ were fit to the following model of Mie scattering in tissue^24^:

1 $${\mu }_{s}^{^{\prime}} \left(\lambda \right)=a{\left(\frac{\lambda }{500nm}\right)}^{-b},$$

to extract parameters $a$, the fitted value of ${\mu }_{s}^{^{\prime}} \left(\lambda =500nm\right)$, and $b$, the “scattering power.” This relationship and assumed values of parameters $a$ and $b$ have been commonly employed to account for the influence of scattering in CW-NIRS techniques^25^. The adjusted R^2^ of the fitted model was also examined to assess the agreement of scattering data with the Mie scattering model at baseline and following asphyxia and CPR.

Independent determination of µ_a_ and µ_s_′ using FD-DOS permitted absolute quantification of [Hb] and [HbO_2_] in cerebral tissue. This was achieved by solving a wavelength-dependent system of equations employing the central relationship:

2 $${\mu }_{a}\left(\lambda \right)={\varepsilon }_{{HbO}_{2}}\left(\lambda \right)\left[{HbO}_{2}\right]+ {\varepsilon }_{Hb}\left(\lambda \right)\left[Hb\right]+0.75{\mu }_{a,{H}_{2}O}\left(\lambda \right),$$

where µ_a_ is the measured absorption coefficient and $\upvarepsilon$ is the known extinction coefficient at wavelength λ^26^. The use of absorption coefficient information at four wavelengths ranging from 690 to 830 nm, compared to the minimum two-wavelength requirement (*e.g.*, 690 and 830 nm)^27^, provided additional constraints to ameliorate the effects of noise when solving for chromophore concentrations. StO_2_ (%) and THC (µmol/L) were then calculated as:

3 $$St{O}_{2} =\frac{\left[Hb{O}_{2}\right]}{\left[Hb{O}_{2}\right]+\left[Hb\right]};$$

4 $$THC =\left[Hb{O}_{2}\right]+\left[Hb\right].$$

### Physiologic monitoring

High-fidelity, solid-state, micromanometer-tipped catheters (Millar Instruments, Houston, TX, USA) were advanced under ultrasound guidance from femoral arterial and venous access points to corresponding thoracic positions for continuous aortic and right atrial pressure sampling (PowerLab; ADInstruments, Australia). Coronary perfusion pressure (CoPP) during CPR was calculated as the difference between the aortic pressure and right atrial pressure during the relaxation phase of chest compressions^28^. Rectal temperature, a 3-lead electrocardiogram, peripheral oxygen saturation, and capnometry were also continuously sampled at a rate of 1 kHz (LabChart 8; ADInstruments, Australia).

### Experimental protocol

**Figure 1:**
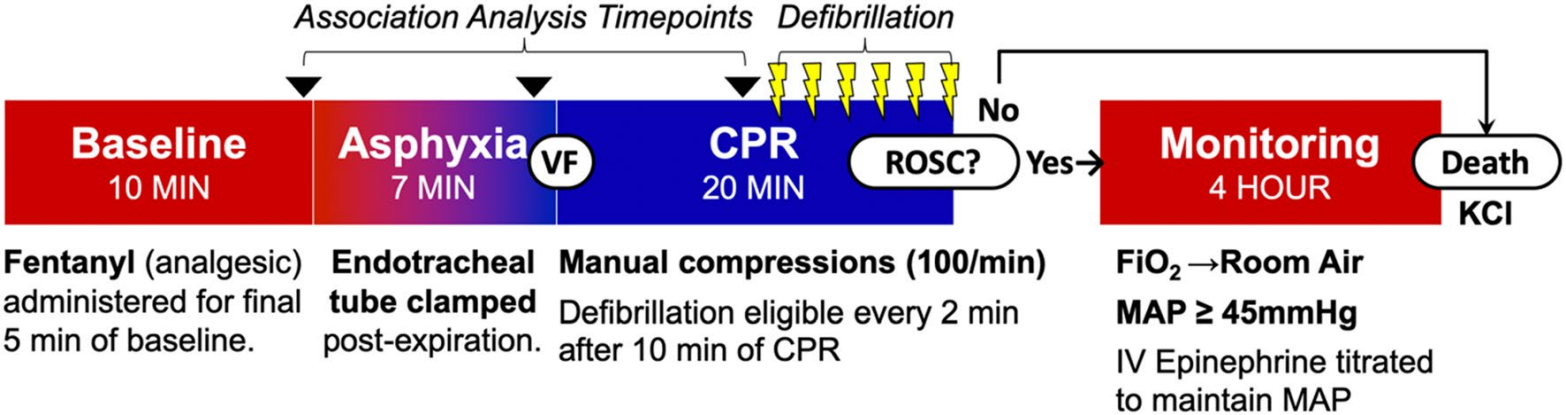
Experimental Protocol: Continuous non-invasive frequency-domain diffuse optical spectroscopy (FD-DOS) measurements of cerebral hemodynamics were continuously acquired from start of Baseline, through Asphyxia, CPR, and four-hours post-Return of Spontaneous Circulation (ROSC). The association between cerebral hemodynamics and ROSC were examined at baseline, the end of asphyxia, and in the 10th minute of CPR prior to the first defibrillation. VF, ventricular fibrillation; BPM, beats per minute; FiO_2_, fraction of inspired oxygen; MAP, mean arterial pressure; KCl, potassium chloride.

All subjects underwent an asphyxia-associated model of cardiac arrest (Fig. 1). Following 7 min of asphyxia, ventricular fibrillation was electrically induced to ensure cardiac arrest. Mechanical ventilation was restarted at a respiratory rate of 10 breaths per minute and, simultaneously, manual chest compressions were initiated at a rate of 100 compressions per minute. Depending on cohort inclusion, subjects were randomized to receive one of the following four CPR strategies:

I. Depth-guided CPR (DG-CPR; n = 14): Resuscitation was conducted in accordance with the 2015 Pediatric Advanced Life Support (PALS) guidelines^29^ with chest compression depths of 1/3 of the anterior–posterior chest diameter, 100% FiO_2_, and epinephrine (0.02 mg/kg IV) every four minutes.
II. DG-CPR with inhaled nitric oxide (DG-CPR + iNO; n = 10): CPR was delivered as in the DG-CPR group with the addition of iNO at 20 parts per million (ppm) beginning one minute into CPR and discontinued at the end of CPR.
III. Hemodynamic-directed CPR with 100% FiO_2_ (HD-CPR 100%; n = 15)^6–10^: Chest compression depth was actively titrated to maintain an a priori aortic systolic pressure goal of either 90 mmHg (n = 13) or 110 mmHg (n = 2). Vasopressors were administered when coronary perfusion pressure fell below 20 mmHg. Vasopressor scheduling followed a three-stage cycle of 0.02 mg/kg of epinephrine, a repeated 0.02 mg/kg of epinephrine, and then a switch to 0.4 units/kg of vasopressin after a minimum interval of one minute after epinephrine doses and two minutes after vasopressin doses.
IV. Hemodynamic-directed CPR with 21% FiO_2_ (HD-CPR 21%; n = 9)^21^: CPR was delivered as in the HD-CPR 100% group except with the use of 21% FiO_2_ during CPR and through 10 min post-ROSC in survivors.

Following ten minutes of chest compressions, subjects became eligible for defibrillation at two-minute intervals. CPR concluded either immediately upon attainment of ROSC or after 20 min of CPR.

### Statistical analysis

Feasibility was assessed by the percentage of subjects where non-invasive FD-DOS data was successfully acquired for the entirety of CPR without data loss. The impact of asphyxia on absolute optical properties was examined by Wilcoxon Signed-Rank test (α = 0.05) comparing baseline values to values at the end of asphyxia. The association of optical properties and cerebral hemodynamic measurements at 10 min of CPR, prior to defibrillation, with the subsequent attainment of ROSC was assessed by Wilcoxon Rank-Sum tests (two-tailed, α = 0.05). These results were used to examine the cerebral physiology associated with high-quality CPR and to identify physiologic parameters for inclusion in ROSC prediction analyses. Physiologic FD-DOS parameters included the absolute values of [HbO_2_], [Hb], StO_2_, and THC, as well as their absolute and relative changes from baseline, and their absolute and relative changes with respect to 1 min into CPR. The 1-min CPR time-point was selected for two reasons. First, this time corresponds to the clinical period allotted for chest molding in swine (*i.e.*, conformational adaptation of the chest wall to the force of compressions). Second, this approach simulated a time delay to allow for placement of non-invasive monitoring after arrest recognition and the initiation of CPR in a clinical scenario.

Baseline values were determined as the mean value in the 2-min prior to asphyxia. Asphyxia-end values were calculated as the mean value acquired in the 15 s prior to the end (*i.e.,* 7 min) of asphyxia. In order to allow 1 min of chest molding and time for FD-DOS placement, 1-min CPR values were calculated as the mean of values measured in the 15 s immediately following 1 min of CPR. The 10-min CPR values were calculated as the mean of values measured in the 10th minute of CPR. Due to the normal distribution of the continuous 10 Hz data collected during these time-frames, they were summarized parametrically. However, the distribution of these summary parameters across subjects in several measured parameters demonstrated a skewed distribution which warranted the use of non-parametric tests, as detailed above, for association analysis.

A post-hoc analysis was conducted to examine the role of CPR strategy as a potential covariate in the relationship between cerebral hemodynamics and ROSC. Fisher’s Exact Test was used to examine the association between CPR strategy and ROSC; pairwise comparison of each alternative CPR strategy group versus the standard, depth-guided (DG-CPR) group was conducted. If a CPR strategy was found to be significantly associated with ROSC, the association between this strategy and each cerebral hemodynamic parameter was examined by pairwise comparison with the DG-CPR group using non-parametric Wilcoxon Rank-Sum tests. Subsequently, the association of cerebral hemodynamics and ROSC was then assessed by both simple and multiple exact logistic regression (controlling for CPR strategy) and compared.

### Selection of optimal predictor and decision threshold

Cerebral hemodynamic parameters significantly associated with ROSC were evaluated for ROSC prediction throughout the first 10 min of CPR. This timing approximates the median duration of CPR in pediatric in-hospital cardiac arrest survivors^13^. Only parameters that could be feasibly measured in an in-hospital cardiac arrest scenario (*i.e.*, after recognition of arrest and start of CPR) were included for prediction analysis; those which were calculated relative to baseline were removed. The resulting parameters comprised absolute values, and absolute and relative changes from 1-min into CPR. The mean value of each parameter from 2 to 10-min of CPR was used to predict ROSC. Predictive performance was quantified by the area under the curve (AUC) of the receiver operating characteristic (ROC) curve, and the weighted Youden index (J_w_), calculated as^30^:

5 $${J}_{w}=2\left(w\times Sensitivity+\left(1-w\right)\times Specificity\right)-1, 0\le w\le 1.$$

Specificity was emphasized in our calculation of J_w_ by assigning a sensitivity weight (*w*) of 0.1 in Eq. (5). The emphasis on specificity minimizes the incidence of false positives within subjects who do not achieve ROSC (*i.e*., false positive rate). It is critical to accurately identify these subjects to enable timely intervention. For each parameter, all measured values were evaluated as potential decision thresholds to predict the outcome of ROSC. The optimal decision threshold was then determined by selecting the value which achieved the highest J_w_. The optimal predictor was then selected by the highest Youden J_w_ achieved across all evaluated parameters.

K-fold cross-validation (k = 10) was performed to assess variability in predictive performance and to assess stability of predictive performance as a function of CPR time. Time-series data were bin-averaged across 1-min intervals from 2 to 10 min of CPR. Within each 1-min interval, predictive performance for each fold was assessed by Youden J_w_ of the test data. Stability over time was confirmed by the absence of significant fluctuations between time intervals of the median value of Youden J_w_ across folds.

## Results

### Feasibility of FD-DOS during CPR

Continuous FD-DOS measurements of absolute optical absorption and scattering properties at wavelengths λ = 690, 725, 785, and 830 nm, and cerebral hemodynamic parameters [HbO_2_], [Hb], StO_2_ and THC were successfully collected throughout asphyxia and CPR in 48/52 subjects (92.3%). In the four excluded subjects, intermittent loss in data quality was directly attributable to motion artifact during CPR whereby light leakage or a shift in probe position resulted in a breakdown of the theoretical semi-infinite photon diffusion model relating source-detector separation to detected light intensity or phase.

**Figure 2:**
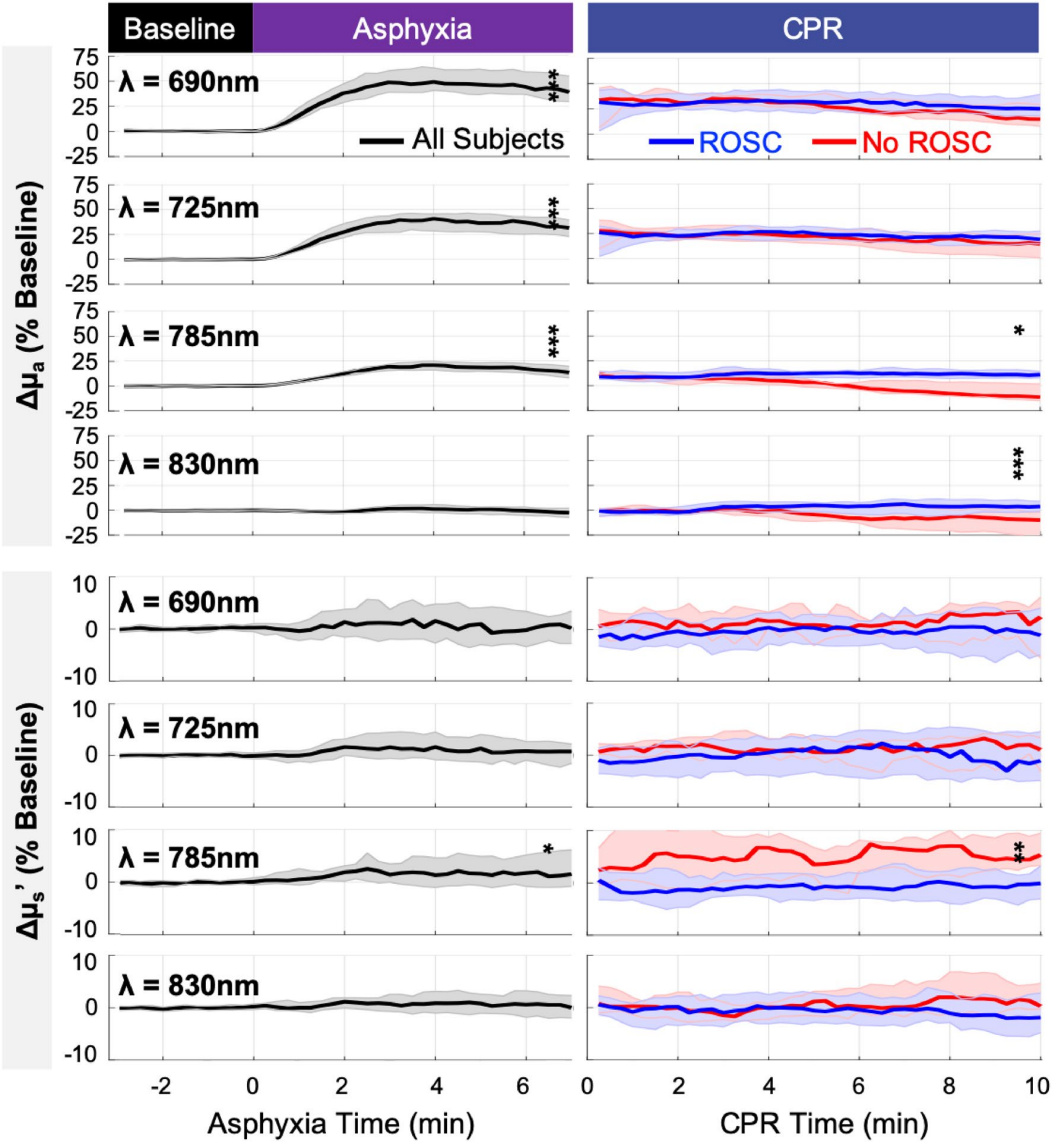
Changes in Optical Absorption (µ_a_) and Scattering (µ_s_′) Properties during Asphyxia (*left*) and CPR (*right*): Shown are the median (*thick line*) and interquartile range (*shaded*) of the measured changes during asphyxia (all subjects, *black*) and during CPR (ROSC, *blue*; No-ROSC, *red*), expressed as a percentage (%) of baseline. Significant increases in µ_a_ at 690, 725, and 785 nm and µ_s_′ at 785 nm were observed at the end of asphyxia. In the 10th minute of CPR, the change in µ_a_ at 785 and 830 nm from baseline was significantly greater in ROSC versus No-ROSC subjects, however change in µ_s_′ at 785 nm was significantly greater in No-ROSC versus ROSC subjects (*, *p* < 0.05; **, *p* < 0.01; ***, *p* < 0.001).

Measurement data are summarized using median and interquartile range in brackets ([IQR]). Baseline optical absorption and scattering properties (µ_a_, µ_s_′) and their changes from baseline during asphyxia and CPR are summarized at key experimental time points in Table 1 and depicted as a function of time in Fig. 2. At the end of asphyxia, with the exception of λ = 830 nm, µ_a_ significantly increased at all wavelengths (*p* < 0.001); µ_a_ (λ = 830 nm) was not significantly impacted (+ 0.00/cm [− 0.01, 0.00], *p* = 0.061). A significant increase in µ_s_′ (λ = 785 nm) was also observed (+ 0.1/cm, *p* = 0.024); significant scattering changes at other wavelengths were not detected. No significant changes were observed in $a$ (the fitted µ_s_′ (500 nm)), $b$ (the scattering power), or the goodness-of-fit of the Mie scattering model. Notably, however, there is high variability in the baseline distribution of these parameters. The IQR of parameter $a$ spans 43.5% of the median; for parameter $b$, the IQR span rises to 81.8% of the median.

**Table 1:** Summary of optical absorption and scattering properties at baseline, end-of-asphyxia and 10-min of CPR.

	Baseline n = 48	Asphyxia, End (Δ Baseline)	*p* value†	CPR, 10 min (Δ Baseline)		
				ROSC n = 37	No-ROSC n = 11	*p* value‡
**Optical absorption coefficient µ**_**a**_** (λ), (1/cm)**						
µ_a_ (690 nm)	0.16 [0.14, 0.19]	+ 0.07 [0.05, 0.09]	< 0.001	+ 0.05 [0.03, 0.06]	+ 0.03 [0.02, 0.05]	0.294
µ_a_ (725 nm)	0.14 [0.12, 0.15]	+ 0.04 [0.03, 0.05]	< 0.001	+ 0.03 [0.02, 0.04]	+ 0.02 [0.00, 0.03]	0.105
µ_a_ (785 nm)	0.14 [0.13, 0.15]	+ 0.02 [0.01, 0.03]	< 0.001	+ 0.01 [0.01, 0.02]	− 0.02 [− 0.02, 0.00]	0.011
µ_a_ (830 nm)	0.15 [0.13, 0.16]	+ 0.00 [− 0.01, 0.00]	0.061	+ 0.00 [− 0.00, 0.01]	− 0.01 [− 0.04, − 0.00]	0.001
**Optical reduced scattering coefficient µ**_**s**_′** (λ), (1/cm)**						
µ_s_′ (690 nm)	10.9 [9.9, 11.8]	+ 0.0 [− 0.4, 0.4]	0.759	− 0.0 [− 0.6, 0.4]	+ 0.3 [− 0.4, 0.6]	0.233
µ_s_′ (725 nm)	10.2 [9.2, 10.9]	+ 0.1 [− 0.2, 0.2]	0.320	− 0.2 [− 0.5, 0.4]	+ 0.1 [− 0.2, 0.4]	0.204
µ_s_′ (785 nm)	9.3 [8.3, 10.2]	+ 0.1 [− 0.1, 0.5]	0.024	− 0.1 [− 0.4, 0.1]	+ 0.5 [0.3, 0.9]	0.005
µ_s_′ (830 nm)	8.7 [7.9, 9.4]	+ 0.0 [− 0.1, 0.2]	0.339	− 0.2 [− 0.5, 0.2]	+ 0.1 [0.0, 0.4]	0.077
**Mie scattering model fit parameters,** µ_s_′(λ) = a*(λ/500 nm)^***b***						
***a***, (1/cm)	15.4 [12.1, 18.8]	+ 0.2 [− 1.3, 1.5]	0.604	− 0.44 [− 1.89, 1.47]	+ 0.59 [− 1.23, 1.63]	0.691
***b***	1.1 [0.7, 1.6]	+ 0.0 [− 0.2, 0.2]	0.837	+ 0.04 [− 0.24, 0.30]	− 0.02 [− 0.20, 0.16]	0.691
Goodness-of-fit (adjusted R^2^)	0.82 [0.54, 0.94]	− 0.02 [− 0.10, 0.05]	0.142	− 0.03 [− 0.22, 0.05]	+ 0.08 [0.01, 0.17]	0.026

Values summarized as median [IQR].

^†^*p* value from Wilcoxon signed-rank test.

^‡^*p* value from Wilcoxon rank-sum test comparing ROSC and No-ROSC groups.

λ, wavelength; Δ Baseline, change from Baseline; ROSC, return of spontaneous circulation; *a*, fitted reduced scattering coefficient at λ = 500 nm; *b*, scattering power.

The change from baseline at 10 min of CPR was compared between subjects who achieved ROSC versus those who did not (No-ROSC). No-ROSC subjects demonstrated significantly smaller changes in µ_a_ at λ = 830 nm (*p* = 0.001) and 785 nm (*p* = 0.011), significantly larger change in µ_s_′ (λ = 785 nm; *p* = 0.005), and, interestingly, a modest improvement in the goodness-of-fit of the Mie scattering model (*p* = 0.026). No other significant differences in optical properties or fitted scattering parameters were observed.

### Association of cerebral hemodynamics and ROSC

**Figure 3:**
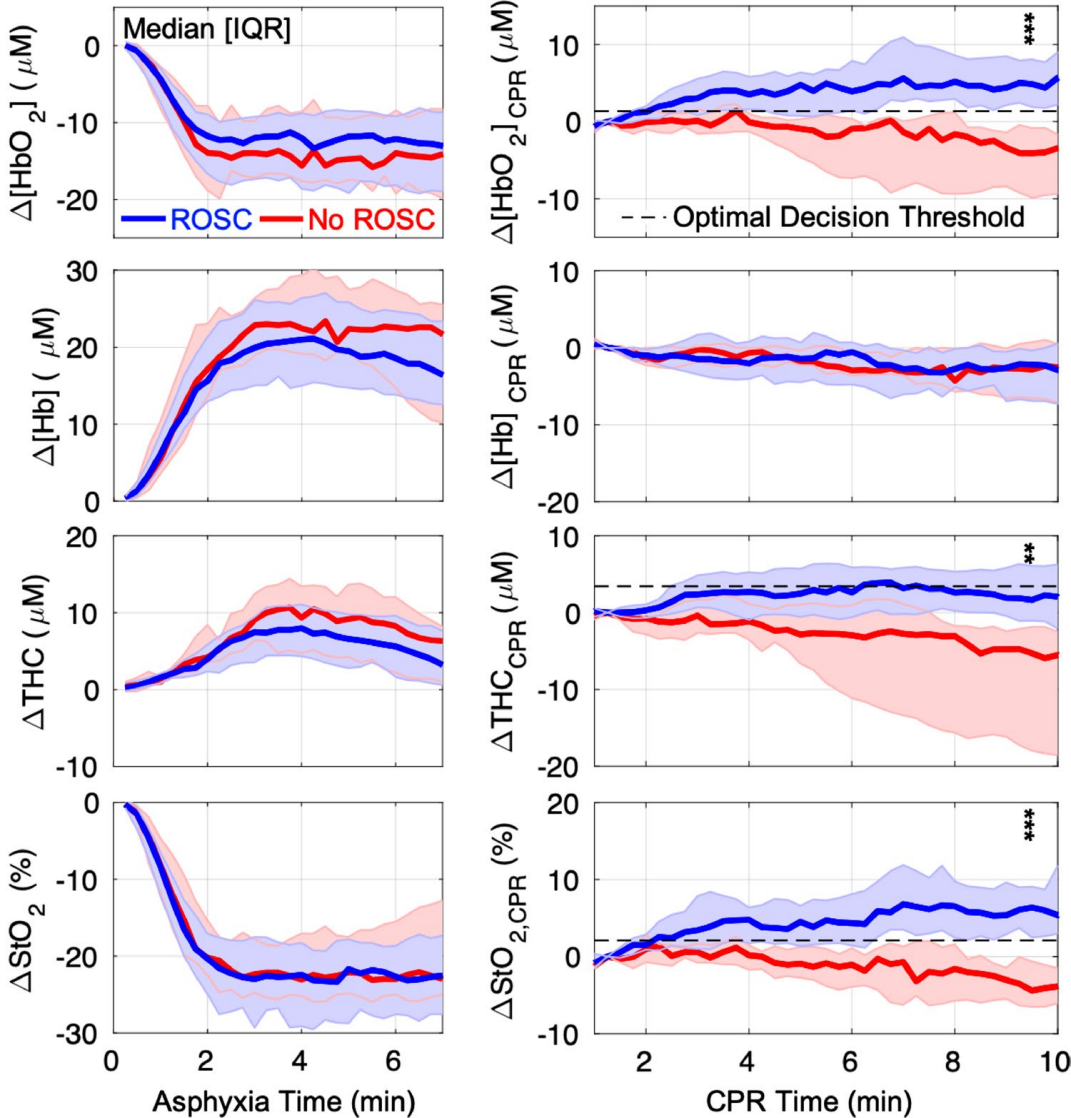
Changes in Cerebral Hemodynamics during Asphyxia and CPR: The changes in cerebral oxy-hemoglobin concentration (Δ[HbO_2_]; *first row*), deoxy-hemoglobin concentration (Δ[Hb]; *second row*), total hemoglobin concentration (ΔTHC; *third row*) and tissue oxygen saturation (ΔStO_2_; *bottom row*) from baseline during asphyxia (*left*), and from 1-min of CPR during CPR (*right*), are summarized and grouped by CPR outcome (ROSC, *blue*; No ROSC, *red*). Data are plotted as median (*thick solid line*) and interquartile range (IQR; *thin solid line*). The optimal decision threshold for ROSC are also plotted (dotted line) for Δ[HbO_2_]_CPR_ (+ 1.3 µmol/L), ΔTHC_CPR_ (+ 3.4 µmol/L), and ΔStO_2,CPR_ (+ 2.1%). In the 10th minute of CPR, association with ROSC was assessed by Wilcoxon Rank-Sum test (**, *p* < 0.01; ***, *p* < 0.001).

A comparison of cerebral hemodynamic parameters between ROSC and No-ROSC groups at baseline, at the end of asphyxia, and at 10 min of CPR, is given in Table 2. No significant differences in cerebral hemodynamics were observed between ROSC and No-ROSC groups at baseline or at the end of asphyxia. However, at 10 min of CPR, subjects with ROSC had higher absolute [HbO_2_] (29.1 [21.7, 33.1] vs. 20.8 [16.1, 23.9] µmol/L, *p* < 0.01), StO_2_ (39.8% [35.1, 44.8] vs. 31.1% [28.0, 35.8], *p* < 0.01), and trended towards higher THC (68.6 [62.9, 78.2] vs. 60.7 [55.5, 71.8] µmol/L, *p* = 0.06). Highly significant differences (*p* < 0.01) between ROSC and No-ROSC groups were also seen in the absolute and relative change from baseline and from 1-min of CPR in all three parameters. No difference between groups was observed for relative or absolute [Hb] values during CPR. Continuous data depicting the absolute change in cerebral hemodynamics during asphyxia (from baseline) and during CPR (from 1-min of CPR) are plotted in Fig. 3.

**Table 2:** Summary of cerebral hemodynamic parameters at baseline, end-of-asphyxia and at 10-min of CPR.

	ROSC, n = 37 Median [IQR]	No-ROSC, n = 11 Median [IQR]	*p* value
**Baseline**			
[HbO_2_], μmol/L	33.4 [29.1, 36.9]	35.1 [28.8, 45.7]	0.317
[Hb], μmol/L	30.9 [26.8, 36.4]	32.4 [30.3, 36.4]	0.443
THC, μmol/L	66.1 [57.5, 69.7]	69.5 [58.9, 76.1]	0.330
StO_2_, %	49.7 [47.4, 56.2]	53.0 [47.8, 55.4]	0.687
**Asphyxia, End**			
[HbO_2_], µmol/L	20.2 [15.6, 24.5]	24.0 [20.3, 25.6]	0.155
[Hb], µmol/L	47.9 [43.3, 56.2]	55.3 [41.3, 57.5]	0.659
THC, µmol/L	66.7 [63.0, 76.0]	78.2 [60.6, 82.8]	0.462
StO_2_, %	28.4 [25.0, 32.3]	31.4 [29.0, 34.8]	0.177
**CPR, 10 min**			
[HbO_2_], µmol/L	29.1 [21.7, 33.1]	20.8 [16.1, 23.9]	0.007
Δ[HbO_2_] from Baseline	− 5.3 [− 11.3, − 1.3]	− 18.5 [− 24.5, − 8.0]	< 0.001
r[HbO_2_] to Baseline, %	84.6 [71.0, 95.6]	52.2 [39.1, 71.9]	0.001
Δ[HbO_2_] from 1 min-CPR	+ 4.4 [2.1, 7.8]	− 3.6 [− 9.5, − 0.7]	< 0.001
r[HbO_2_] to 1 min-CPR, %	120.0 [110.5, 141.1]	87.0 [63.3, 95.4]	< 0.001
[Hb], µmol/L	41.7 [37.8, 47.1]	43.8 [37.1, 49.6]	0.477
Δ[Hb] from Baseline	+ 9.8 [7.2, 13.7]	+ 11.0 [6.5, 17.2]	0.745
r[Hb] to Baseline, %	131.2 [121.5, 145.2]	127.5 [122.2, 153.1]	0.845
Δ[Hb] from 1 min-CPR	− 2.3 [− 7.0, − 0.2]	− 2.6 [− 6.7, 0.5]	1.000
r[Hb] to 1 min-CPR, %	94.3 [86.0, 99.6]	93.0 [86.8, 102.0]	0.825
StO_2_, %	39.8 [35.1, 44.8]	31.1 [28.0, 35.8]	0.003
ΔStO_2_ from Baseline	− 12.4 [− 16.2, − 5.6]	− 23.5 [− 27.0, − 13.5]	0.004
rStO_2_ to Baseline, %	76.0 [68.9, 89.8]	54.8 [50.0, 74.3]	0.003
ΔStO_2_ from 1 min-CPR	+ 6.6 [3.4, 9.1]	− 3.9 [− 6.5, − 0.8]	< 0.001
rStO_2_ to 1 min-CPR, %	118.1 [108.9, 130.6]	90.4 [80.5, 96.4]	< 0.001
THC, µmol/L	68.6 [62.9, 78.2]	60.7 [55.5, 71.8]	0.059
ΔTHC from Baseline	+ 6.1 [2.3, 9.4]	− 2.3 [− 15.6, 3.8]	0.002
rTHC to Baseline, %	109.6 [103.6, 115.0]	95.9 [78.9, 105.3]	0.001
ΔTHC from 1 min-CPR	+ 1.7 [− 1.3, 6.1]	− 5.4 [− 18.0, − 2.1]	0.001
rTHC to 1 min-CPR, %	102.5 [98.0, 108.9]	93.1 [78.6, 96.9]	0.002

[HbO_2_], oxy-hemoglobin concentration; [Hb], deoxy-hemoglobin concentration; StO_2_, tissue oxygen saturation; THC, total hemoglobin concentration; Δ-prefix, absolute change; r-prefix, relative value compared to 100% at baseline.

Our observational data collection included subjects who underwent one of four different CPR strategies: DG-CPR, DG-CPR + iNO, HD-CPR 100% and HD-CPR 21% (see “Methods” for a detailed explanation of these strategies). Tabular results of a post-hoc analysis examining the influence of CPR strategy on the association of cerebral hemodynamics and ROSC are included in Supplementary Materials. Examining the association of CPR strategy and ROSC, the DG-CPR + iNO group (100% ROSC) trended towards significantly greater incidence of ROSC versus the DG-CPR group (64.3% ROSC; one-sided *p* = 0.047; see Supplementary Table S1). Note, only a one-sided *p* value could be computed using Fisher’s Exact Test due to the lack of No-ROSC subjects in the DG-CPR + iNO group. Subsequent comparison of cerebral hemodynamic parameters between the DG-CPR + iNO group and the DG-CPR group did not identify a significant association between CPR strategy and any cerebral hemodynamic parameter (Supplementary Table S2). Comparing simple to multiple logistic regression models incorporating DG-CPR and DG-CPR + iNO groups, CPR strategy did not impact the relationship between each parameter and ROSC (Supplementary Table S3); no clinically significant difference was observed in the odds ratios nor their respective *p* values. All significant hemodynamic parameters identified by rank-sum test (absolute [HbO_2_] and StO_2_ as well as the relative and absolute changes from baseline and 1-min of CPR of [HbO_2_], StO_2_, and THC) were also found to have a significant model effect in logistic regression models. This demonstrates that CPR strategy did not impact the relationship between each parameter and ROSC.

### Selection of optimal predictor and decision threshold

**Figure 4:**
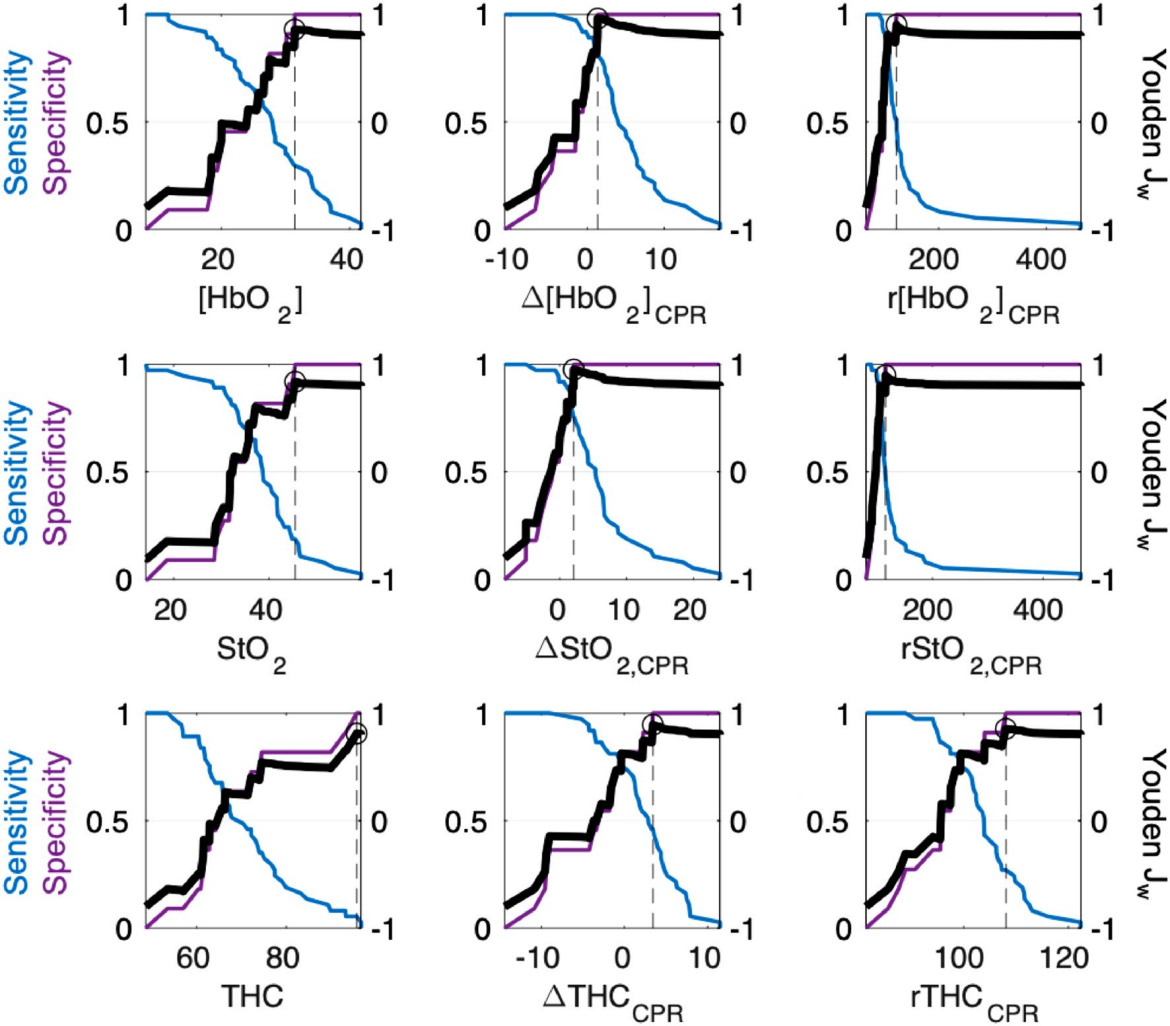
Selection of Optimal Predictor and Decision Threshold Based on Maximum Weighted Youden Index (J_w_): From the mean value from 2 to 10 min of CPR, the optimal decision threshold was selected by maximum ROSC prediction performance, as assessed by the weighted Youden index (J_w_). For each parameter, the sensitivity (*blue*, left y-axis) and specificity (*purple*, left y-axis) for ROSC, and the calculated Youden J_w_ (*bold black line*, right y-axis) are plotted for all possible threshold values (x-axis). The parameter-specific maximum J_w_ is highlighted by a circle with a dotted line drawn to the corresponding threshold value on the x-axis; these values are listed in Table 3. Δ[HbO_2_]_CPR_ achieved the highest J_w_ (0.96) overall; this corresponds to a decision threshold of + 1.3 µmol/L.

The optimal ROSC predictor and its decision threshold were determined from the mean value from 2 to 10-min of CPR. In Fig. 4, the corresponding sensitivity, specificity and J_w_ for all possible decision threshold values are shown for each evaluated parameter. An additional depiction of the ROC curve and calculated AUC is shown in Supplementary Fig. S1. In Table 3, we list these AUC values, the max Youden J_w_ achieved, and the corresponding decision threshold. Additionally, we report the minimum and maximum decision thresholds and the median and interquartile range ([IQR]) of J_w_ from tenfold cross-validation analysis. To assist in the interpretation of J_w_, it is important to note that, with a sensitivity weighting w = 0.1, it is possible to achieve a J_w_ = 0.8 for any predictor by selecting an arbitrarily high decision threshold that renders a specificity of 1 and a sensitivity of 0; this can be seen in each sub-panel in Fig. 4. While lower values of J_w_ may be achieved for arbitrary decision thresholds, the maximum J_w_ for an individual AUC curve will be, at minimum, 0.8. More optimal decision thresholds which preserve sensitivity increase J_w_ to a maximum value of 1 when both sensitivity and specificity are 1.

**Table 3:** Predictive performance and optimal decision threshold for significant cerebral hemodynamic parameters.

Significant parameters	AUC (95% CI)	J_w_	Decision threshold	10-fold cross-validation	
				Decision threshold [Min, Max]	J_w_ Median [IQR]
[HbO_2_], µmol/L	0.68 (0.50, 0.82)	0.86	31.4	[30.2, 33.4]	0.83 [0.80, 0.87]
Δ[HbO_2_] from 1 min-CPR	0.94 (0.83, 0.98)	0.96	+ 1.3	[+ 1.3, + 1.9]	0.95 [0.93, 1.00]
r[HbO_2_] to 1 min-CPR, %	0.91 (0.76, 0.97)	0.90	121.5	[105.5, 121.9]	0.90 [0.87, 0.90]
StO_2_, %	0.70 (0.50, 0.85)	0.84	45.5	[44.2, 46.2]	0.80 [0.80, 0.83]
ΔStO_2_ from 1 min-CPR	0.92 (0.81, 0.97)	0.95	+ 2.1	[+ 1.9, + 2.4]	0.94 [0.93, 0.95]
rStO_2_ to 1 min-CPR, %	0.90 (0.74, 0.97)	0.90	114.7	[105.3, 115.3]	0.87 [0.80, 0.93]
THC, µmol/L	0.59 (0.38, 0.78)	0.81	95.8	[93.2, 95.8]	0.80 [0.80, 0.80]
ΔTHC from 1 min-CPR	0.83 (0.66, 0.93)	0.89	+ 3.4	[+ 2.3, + 3.6]	0.88 [0.86, 0.94]
rTHC to 1 min-CPR, %	0.80 (0.60, 0.91)	0.85	108.1	[104.0, 109.6]	0.80 [0.38, 0.82]

AUC = area under curve of receiver-operating-characteristic; J_w_ = maximum weighted Youden J index; [HbO_2_] = oxy-hemoglobin concentration; StO_2_ = tissue oxygen saturation; THC = total hemoglobin concentration; Δ-prefix = absolute change; r-prefix = relative value compared to 100% at baseline.

Overall, absolute change in [HbO_2_] from 1-min of CPR (Δ[HbO_2_]_CPR_) achieved the highest J_w_ of 0.96 and the highest AUC of 0.94. This J_w_ corresponded to an optimal decision threshold of Δ[HbO_2_]_CPR_ =  + 1.3 µmol/L (Fig. 4). The absolute change in StO_2_ from 1-min of CPR (ΔStO_2,CPR_) had similar predictive value (J_w_ = 0.95, AUC = 0.92). Cross-validation analysis confirmed our findings; the tenfold distribution of J_w_ for ΔStO_2,CPR_ (J_w_ = 0.94 [0.93, 0.95]) trended lower, but maintained overlap with that of Δ[HbO_2_]_CPR_ (J_w_ = 0.95 [0.93, 1.00]).

**Figure 5:**
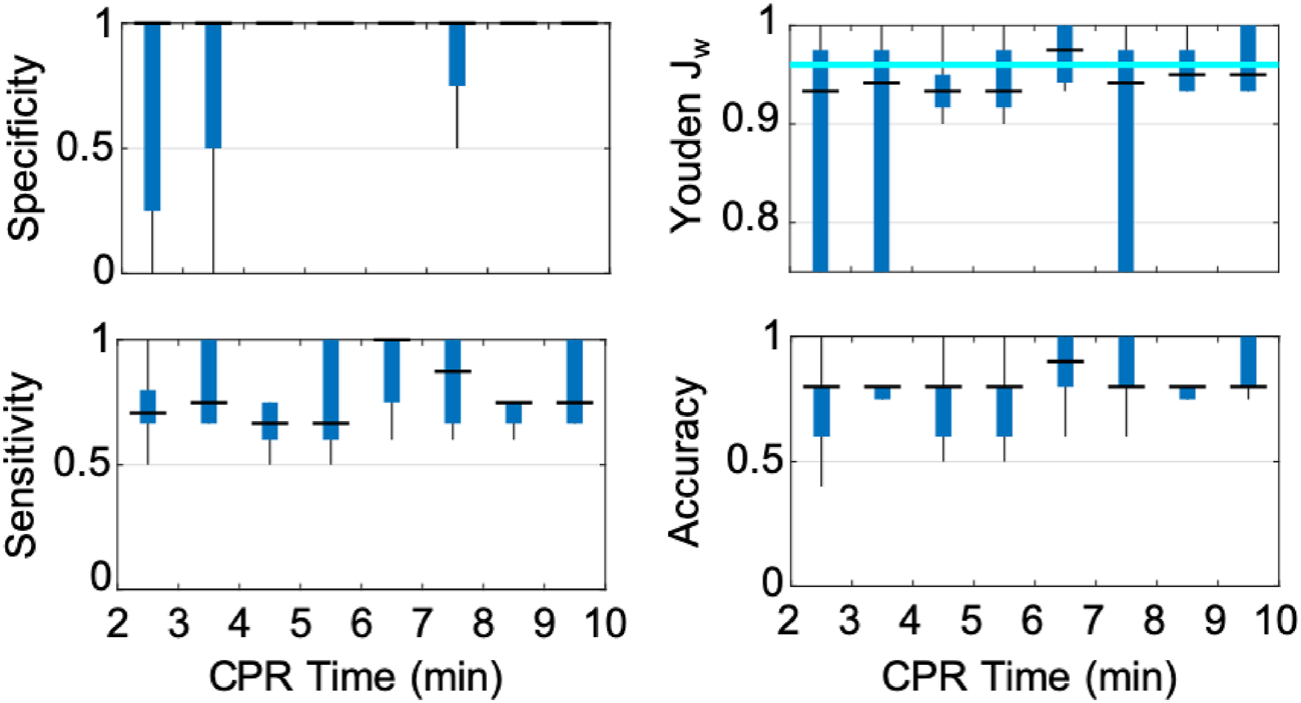
Time-Dependent Prediction Performance during Early CPR: The predictive performance of the optimal decision threshold (Δ[HbO_2_]_CPR_ ≥  + 1.3 µmol/L), across 10 independent test folds, is summarized (median, *horizontal line*; interquartile range, *box*; range, *whisker*) minute-to-minute from 2 to 10 min of CPR. Within each 1-min interval, the decision threshold was applied to predict ROSC in each test fold and the corresponding specificity (top left), sensitivity (bottom left), accuracy (bottom right), and J_w_ (top right) were calculated. This provides a realistic rendering of the variability and time-dependence of the predictive performance of Δ[HbO_2_]_CPR_. For comparison, the parameter’s max J_w_ derived from mean 2–10 min CPR values, is overlaid in the J_w_ sub-panel as a horizontal cyan line. A median specificity of 1, median sensitivity > 0.5, and a median Youden J_w_ > 0.9 is achieved at all time points.

In Fig. 5, we depict the time-dependent variability in ROSC prediction performance of Δ[HbO_2_]_CPR_ during the first 10 min of CPR. By applying the parameter’s optimal decision threshold for ROSC (Δ[HbO_2_]_CPR_ ≥  + 1.3 µmol/L) to each of the tenfold test groups, in 1-min time intervals, we generated a realistic distribution of the Youden J_w_. From 2 to 10 min of CPR, Δ[HbO_2_]_CPR_ ROSC prediction sustained a median specificity of 1, median sensitivity > 0.5, and a median J_w_ > 0.90. This demonstration of stability supports the feasibility of a static decision threshold that may be applied early and throughout the first 10 min of CPR with consistent predictive performance. Time-dependent variability of J_w_ for the optimal decision threshold of each evaluated parameters is shown in Supplementary Fig. S2. In Supplementary Fig. S3, we include additional time-dependent plots of specificity, sensitivity, and accuracy for comparison of Δ[HbO_2_]_CPR_ to ΔStO_2,CPR_ and StO_2_ as these parameters may be interpreted similarly to more commonly reported CW-NIRS metrics of rSO_2_ or TOI.

## Discussion

In this work, cerebral hemodynamic data from quantitative, non-invasive frequency-domain diffuse optical (FD-DOS) monitoring during CPR exhibited excellent *early* predictive utility for ROSC in a pediatric swine model of asphyxia-associated cardiac arrest. Using the optimal decision threshold (Δ[HbO_2_]_CPR_ =  + 1.3 µmol/L), robust, highly specific prediction of ROSC with preserved sensitivity (Youden J_w_ > 0.9, *w* = 0.1) was achieved in the third minute of CPR and maintained throughout early CPR (< 10 min). These findings represent a critical step towards development of a precise, non-invasive CPR quality metric based on cerebral oxygenation and hemodynamics during CPR to optimize outcomes.

The ability of a non-invasive physiologic monitor to discriminate survivors from non-survivors early in the course of CPR has broad potential implications on resuscitation care. Sub-optimal values could trigger the clinical team to improve CPR mechanics (*e.g.,* rate, depth), to employ different vasopressor strategies, or to decide upon extracorporeal support at a much earlier stage. While coronary perfusion pressure (CoPP), diastolic blood pressure, and end-tidal carbon dioxide are physiologic measures of CPR quality that are associated with ROSC, FD-DOS is easily applied and does not require an invasive arterial catheter or an invasive airway. Therefore, this technology has the potential to extend intra-arrest physiologic monitoring and prognostication to a broader population. Moreover, the measurement of cerebral physiology as opposed to, or in addition to, systemic physiology could allow for the optimization of cerebral hemodynamics to improve neurologic outcomes^6–9^.

The association of cerebral oxygenation with ROSC in this study is not surprising—oxygen delivery to the brain during CPR results from adequate oxygenation of blood and adequate cerebral perfusion pressure. Cerebral perfusion pressure and its determinants (*e.g.*, mean arterial pressure) have been shown to be highly correlated with CoPP during CPR, because diastolic blood pressure is both the driving force of CoPP and a key determinant of mean arterial pressure^31,32^. Given the established association between CoPP and ROSC^33^, it is likely that CPR which provides adequate CoPP for oxygen delivery to the heart also provides adequate cerebral perfusion for oxygen delivery to the brain. What is striking, however, is how rapidly [HbO_2_] is able to discern the outcome of ROSC during CPR. No significant differences in [HbO_2_] or its change are present between the ROSC and No-ROSC groups at the end of asphyxia; however, by the third minute of CPR, a change in [HbO_2_] from 1-min into CPR is able to achieve an AUC of 0.82. Thus, change in [HbO_2_] is a promising rapid and specific non-invasive metric of CPR quality.

We also observed a decrease in THC during CPR in No-ROSC subjects, though specificity of ROSC prediction was inferior to [HbO_2_] and StO_2_. Decreases in the blood volume of the microvascular space, as reflected by a decrease in measured THC^34^, may be linked to specific stages of the hypoxic-ischemic response resulting in progressive cerebral edema, microvascular thrombosis, and/or vasospasm. Hypoxia-associated osmotic imbalance combined with complex interactions in the neurovascular bundle promote progressive intracellular derangement and swelling of astrocytic foot processes which encircle all microvasculature^35–37^. Declining THC signal (see Fig. 3) may reflect increasing microvascular compression, which has been transiently observed after temporary cerebral ischemia^37^. Following onset of cardiac arrest, the brain is reliant on effective CPR to combat this pathophysiologic process. Without adequate cerebral perfusion pressure, increasing intracranial pressure may cause further microvascular compression, stagnation of red blood cells in capillaries and post-capillary venules, microthrombi formation and potentially irreversible endothelial damage^36^. The magnitude of THC change in the peri-arrest period has been associated with histopathological cortical injury following resuscitation after extended hypoxic-ischemia in newborn piglets^38^. Thus, THC may serve as an important real-time marker of microvascular integrity in the peri-arrest period with implications for neurological outcomes post-ROSC. Further work is necessary to understand individual susceptibility to microvascular derangement and how THC may be modified during cardiac arrest; however, these non-invasive optical techniques should aid in understanding this complicated pathophysiology.

Recent studies have made significant strides in assessing the predictive value of non-invasive CW-NIRS monitoring of cerebral oxygen saturation (rSO_2_) for ROSC during the conduct of in-hospital resuscitation in adults^15,16,39^. These studies reported similar observations of improved rSO2 changes in patients who achieved ROSC. Our novel application of FD-DOS to CPR in the present work offers important improvements in physiologic quantification that may enhance predictive value of HbO_2_ and StO_2_ compared to CW-NIRS rSO_2_. Using independent measurements of optical scattering (µ_s_′) at multiple wavelengths, we were able to evaluate the goodness-of-fit and distribution of Mie scattering model parameters which are employed in CW-NIRS techniques to account for the magnitude (i.e., parameter “a”) and wavelength-dependence (i.e., parameter “b”; *see* Eq. 1) of scattering; these are commonly assumed and assumed to remain constant with respect to time in CW-NIRS techniques. Our data cautions against the use of population-based assumptions; at baseline, we observed wide inter-subject variability in parameters $a$ and $b$ with coefficient of variations of ~ 30% and ~ 60%, respectively. Furthermore, change from baseline in µ_s_′ (785 nm) (+ 0.5/cm [0.3, 0.9] vs. − 0.1 [− 0.4, 0.1]) and the goodness-of-fit of the Mie scattering model (+ 0.08 [0.01, 0.17] vs. − 0.03 [− 0.22, 0.05]) was significantly greater in subjects who did not achieve ROSC versus those who did. This significant 5–10% change in scattering is consistent with previous observations following euthanasia by pentobarbital in neonatal pigs^40^ and during extreme hypoxia (sagittal sinus oxygen saturation < 10%)^41^. In the setting of profound ischemia, while dynamic spectral scattering characteristics remain poorly defined, changes in scattering at specific wavelengths (*e.g.*, 758 nm, 830 nm) have been shown to become significant^22,40^, with the Fahraeus effect as a potential mechanism whereby decreased axial alignment at lower red blood cell flow velocities cause dynamic hemoconcentration within the vessel and increased scattering^42^ Increasing platelet deposition and aggregation may also contribute to our findings^43,44^. The ability to capture the presence of spectral scattering features of neurological injury which deviate from the Mie scattering model is a critical advantage of FD-DOS over CW-NIRS.

We have additionally implemented the weighted Youden index, cross-validation, and time-dependent k-fold analysis to incorporate quantitative trade-offs between sensitive and specific ROSC prediction for threshold determination, and to assess the stability and time-dependence of predictive performance throughout the resuscitation period. Emphasis on the specificity of ROSC prediction within our metric of predictive performance (Youden J_w_) resulted in the selection of optimal decision thresholds that minimize the rate of false-positive prediction. When applied to real-time resuscitation guidance, this prioritizes identification of subjects who require additional intervention to achieve ROSC. Cerebral hemodynamic parameters that also preserved sensitivity to ROSC achieved the most optimal Youden index. Thus, unnecessary intervention is also minimized. By cross-validating predictive performance and confirming the stability of this performance with respect to CPR time, we are increasingly confident that Δ[HbO_2_]_CPR_ may be used to identify, in the first few minutes, whether ongoing CPR sufficiently supports the hemodynamic needs of the patient. In the future, direct comparison with CW-NIRS is needed to determine whether FD-DOS quantification indeed improves prediction.

Open questions remain regarding appropriate interventions to treat deficits in Δ[HbO_2_]_CPR_ during CPR. A promising strategy drawn from hemodynamic-directed CPR may be titration of compression depth or vasopressor administration. However, given the myriad physiologic predictors of ROSC currently reported^1^, whether or not FD-DOS monitoring of Δ[HbO_2_] will further improve existing management strategies may vary with clinical setting, and availability and feasibility of alternative physiologic monitors. While we did not observe an association between CPR strategy and cerebral hemodynamics, this analysis was limited by small sample sizes (< 10) within strategy; a larger study should be performed to test this association. If an association is found, the associated strategy could potentially be used as an intervention to improve cerebral hemodynamics during CPR.

Numerous experimental considerations were implemented to facilitate clinical translation. An asphyxial injury was selected to model the predominant respiratory-mediated etiology of pediatric in-hospital arrests^13,45^. The relative comparability of chest mechanics^46^ and brain size, development and physiology^47,48^ of pediatric swine motivated their use, by our group and others, for CPR studies of neurological injury^6–11,49,50^, as well as in FD-DOS validation studies^22,23,41^. A neonatal swine model of post-hypoxic encephalopathy^51^ demonstrated similar clinical, electrophysiological, and neuropathological findings to term infants suggesting comparable physiologic response and injury susceptibility to hypoxia. In the present work, precise protocolized maintenance of age-appropriate, normative physiologic values (*e.g.*, pCO_2_, temperature, blood pressure)^52,53^ at baseline minimized inter-subject variability and helped to ensure a healthy neurovascular response to hypoxia and ischemia. The anesthetics administered prior to and during asphyxia (isoflurane and intravenous fentanyl, respectively), have both been shown to decrease cerebral metabolism^54^, which may convey a neuroprotective effect in the setting of hypoxic-ischemic injury^55^. However, isoflurane is also known to impact cardiac contractility and decrease cardiac output and stroke volume in children^56^. To limit this effect, isoflurane dosing was consistently titrated to the minimum necessary to maintain sedation. Clinical variation in comorbidities, arrest etiologies (*e.g.*, cardiac, trauma) and duration of asphyxia and cardiac arrest prior to initiation of CPR may also alter both predictive utility and optimal decision thresholds. Independent, prospective validation in humans is necessary to confirm our findings and to establish appropriate interventions and use case scenarios for CPR optimization using non-invasive neuromonitoring.

Advanced, quantitative, non-invasive diffuse optical neuromonitoring was feasible to apply during CPR and associations between measured cerebral hemodynamics and ROSC were determined in a relevant model of asphyxia-associated pediatric in-hospital cardiac arrest. Our results provide novel insights to the cerebral physiology of resuscitation and propose new, promising non-invasive predictors of ROSC that may be utilized within minutes of CPR initiation to optimize outcomes. Using the optimal decision threshold (Δ[HbO_2_]_CPR_ =  + 1.3 µmol/L), robust, highly specific prediction of ROSC with preserved sensitivity (Youden J_w_ > 0.9, w = 0.1) was achieved within the first few minutes of CPR and maintained throughout early CPR (< 10 min). Further clinical study of non-invasive hemodynamic neuromonitoring during cardiac arrest and resuscitation is needed.

## Supplementary Information

Supplementary Information

## Data Availability

All experimental datasets, analyses, and abstracted parameters reported are available from the corresponding author upon reasonable request.
