# Supplementary material for: Non-invasive diffuse optical neuromonitoring during cardiopulmonary resuscitation predicts return of spontaneous circulation
Source: Sci Rep. 2021 Feb 15;11:3828. doi: 10.1038/s41598-021-83270-5 (PMC7884428; doi:10.1038/s41598-021-83270-5)
Supplement: Supplementary file 1 — Supplementary Information [file 41598_2021_83270_MOESM1_ESM.pdf]

Non-invasive diffuse optical neuromonitoring during cardiopulmonary resuscitation predicts return of spontaneous circulation

Tiffany S. Ko, Constantine D. Mavroudis, Ryan W. Morgan, Wesley B. Baker, Alexandra M. Marquez, Timothy W. Boorady, Mahima Devarajan, Yuxi Lin, Anna L. Roberts, William P. Landis, Kobina Mensah-Brown, Vinay M. Nadkarni, Robert A. Berg, Robert M. Sutton, Arjun G. Yodh, Daniel J. Licht, Wensheng Guo, and Todd J. Kilbaugh

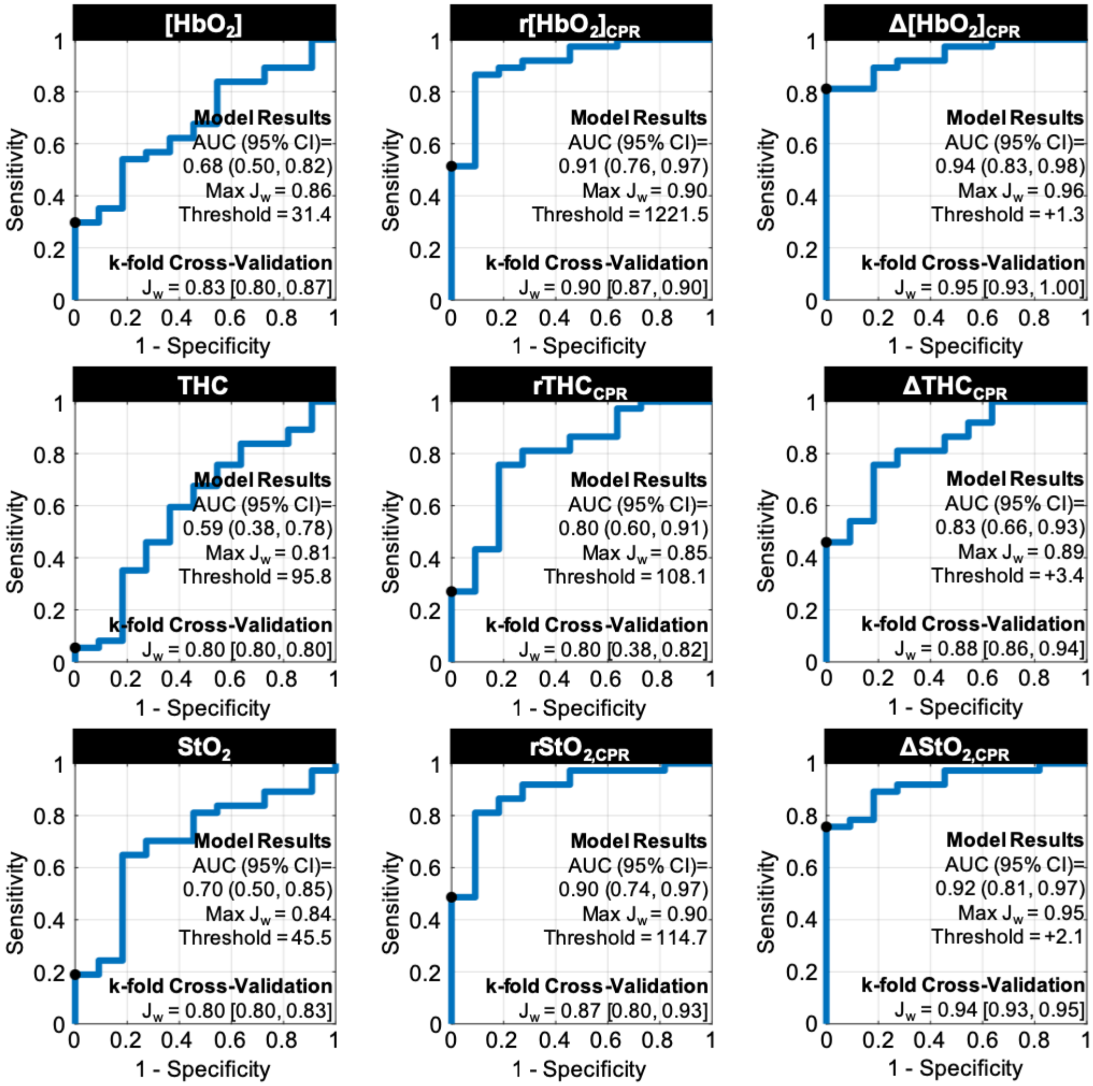

Supplementary Figure S1. ROC Curves for Prediction of ROSC: Receiver operating characteristic (ROC) curves for the prediction of ROSC are depicted in individual sub-panels for each cerebral hemodynamic parameter. The point on the ROC curve corresponding to the maximum weight Youden index (J_w) and optimal decision threshold is marked (black dot). These values are listed within the sub-panel alongside the AUC of the ROC curve and the distribution of J_w values resulting from k-fold (k=10) cross validation. The optimal predictor, $\Delta[HbO_2]_{CPR}$, achieved an AUC of 0.94 and a J_w of 0.96, corresponding to a decision threshold of $\Delta[HbO_2]_{CPR}$ = +1.3µmol/L.

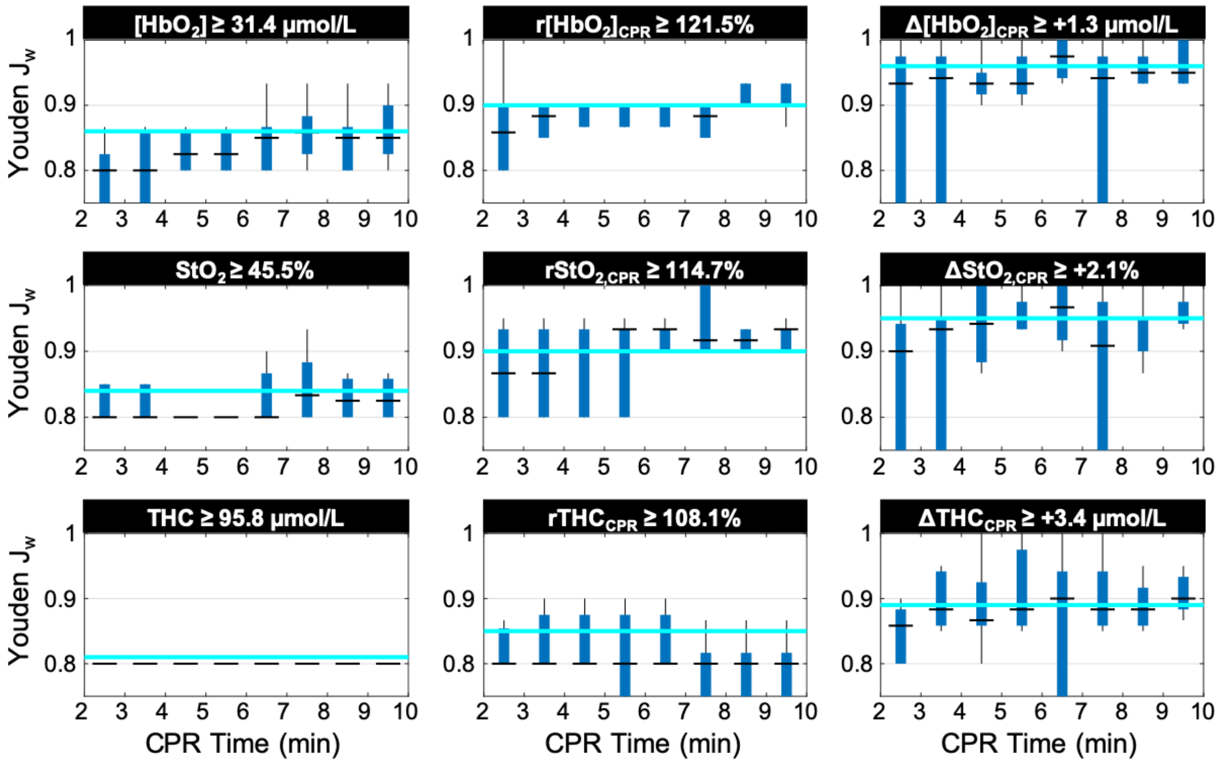

Supplementary Figure S2. Time-Dependent Prediction Performance and Variability during Early CPR: The predictive performance (J_W) of the optimal decision threshold (sub-panel title), across 10 test folds, is summarized (median, *horizontal line*; interquartile range, *box*; range, *whisker*) minute-to-minute from two to 10 minutes of CPR, for each cerebral hemodynamic parameter. Within each 1-minute interval, the decision threshold was applied to predict ROSC in each test fold and a J_W was calculated from the sensitivity and specificity of prediction. This provides a realistic rendering of the variability and time-dependence of each parameter's predictive performance. For comparison, the parameter's max J_W derived from the original predictive model trained on mean 2-10min CPR data, is overlaid as a horizontal cyan line.

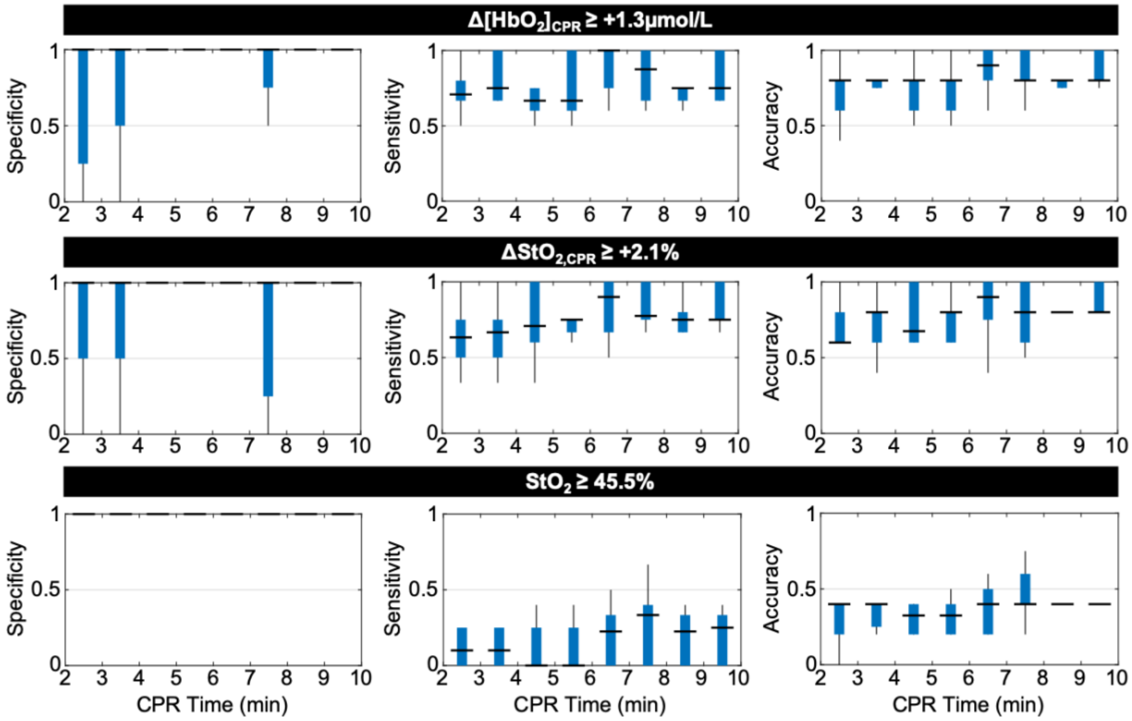

Supplementary Figure S3. Time-Dependent 10-fold Decision Threshold Performance during Early CPR – Specificity, Sensitivity and Accuracy: Prediction performance metrics of specificity (*left*), sensitivity (*center*), and accuracy (*right*) are depicted for change in $[HbO_2]$ and change in StO_2 from 1-minute of CPR ($\Delta[HbO_2]_{CPR}$, *top row*; $\Delta StO_{2,CPR}$, *middle row*) and absolute value of StO_2 (*bottom row*) during the first 10 minutes of CPR. Performance was assessed in and summarized across 10 test folds in 1-minute intervals. Superior sensitivity and accuracy are achieved at all CPR time points by change (Δ) parameters compared to absolute StO_2.

Supplementary Table S1. Effect of CPR Strategy on ROSC, Comparison with Depth-Guided CPR

**CPR Strategy**	**No ROSC**, n	**ROSC**, n	**Odds Ratio** (95% CI)	**p-value**
DG-CPR (ref)	5	9	-	-
DG-CPR+iNO	0	10	6.41 (1.03, ∞)*	0.047*
HD-CPR 100%	5	10	1.21 (0.21, 7.26)	1.000
HD-CPR 21%	1	8	4.19 (0.35, 236.0)	0.417

*Median unbiased estimate with one-sided p-value due to absence of “No ROSC” subjects.
Definition of abbreviations: ref = reference group; DG-CPR = depth-guided CPR strategy; DG-CPR+iNO = depth-guided CPR with inhaled nitric oxide; HD-CPR 100% = hemodynamic-directed CPR with 100% fraction of inhaled oxygen; HD-CPR 21% = hemodynamic-directed CPR with 21% fraction of inhaled oxygen

Supplementary Table S2. Effect of CPR Strategy on Cerebral Hemodynamics, Comparison with Depth-Guided CPR

	**DG-CPR**<br>n=14<br>Median [IQR]	**DG-CPR+iNO**<br>n=10<br>Median [IQR]	**p-value**
**CPR, 10 minutes**			
[HbO_2], µmol/L	22.3 [17.7, 28.7]	27.1 [21.3,35.3]	0.266
Δ[HbO_2] from Baseline	-10.2 [-20.0, -4.2]	-5.7 [-11.5, -1.7]	0.292
r[HbO_2] to Baseline, %	69.2 [40.2, 81.6]	86.1 [55.9, 92.8]	0.292
Δ[HbO_2] from 1min-CPR	+3.0 [-1.3, 7.5]	+7.4 [2.1, 8.8]	0.292
r[HbO_2] to 1min-CPR, %	114.3 [92.4, 135.7]	133.3 [109.7, 146.2]	0.320
StO_2, %	30.8 [27.8, 39.0]	41.3 [36.1, 48.0]	0.143
ΔStO_2 from Baseline	-15.6 [-26.8, -9.1]	-15.3 [-24.0, -5.4]	0.598
rStO_2 to Baseline, %	66.5 [50.0, 80.2]	74.2 [58.9, 88.2]	0.447
ΔStO_2 from 1min-CPR	+3.3 [-1.1, +7.3]	+7.5 [3.6, 13.9]	0.219
rStO_2 to 1min-CPR, %	110.8 [91.1, 121.7]	125.8 [111.1,142.6]	0.349
THC, µmol/L	65.0 [61.5, 76.8]	67.1 [62.1, 73.5]	0.725
ΔTHC from Baseline	+3.2 [-3.2, 6.6]	+6.1 [2.6, 9.1]	0.266
rTHC to Baseline, %	105.5 [95.3, 110.0]	111.1 [105.2, 114.6]	0.160
ΔTHC from 1min-CPR	+1.0 [-4.9, 6.1]	+1.4 [-3.1, 3.5]	0.725
rTHC to 1min-CPR, %	101.6 [93.6, 108.7]	102.0 [94.9, 107.1]	0.725

Definition of abbreviations: DG-CPR = depth-guided CPR strategy; DG-CPR+iNO = depth-guided CPR with inhaled nitric oxide; [HbO_2] = oxy-hemoglobin concentration; [Hb] = deoxy-hemoglobin concentration; StO_2 = tissue oxygen saturation; THC = total hemoglobin concentration; Δ-prefix = absolute change; r-prefix = relative value compared to 100% at baseline.

Supplementary Table S3. Odds Ratio (OR) and Standard Error (SE) of ROSC for Individual Cerebral Hemodynamic Parameters at 10-Minutes of CPR, Adjusted by CPR Strategy

	**Unadjusted**		**Adjusted**	
	**OR** (95% CI)	**p-value**	**OR** (95% CI)	**p-value**
[HbO_2], µmol/L	1.14 (1.01, 1.34)	0.040	1.19 (1.00,1.55)	0.053
Δ[HbO_2] from Baseline	1.31 (1.08, 1.79)	<0.001	1.59 (1.08, 8.20)	0.002
r[HbO_2] to Baseline, %	1.10 (1.02,1.21)	0.002	1.12 (1.03,1.31)	0.003
Δ[HbO_2] from 1min-CPR	1.73 (1.14, 4.70)	<0.001	2.13 (1.19, ∞)	<0.001*
r[HbO_2] to 1min-CPR, %	1.11 (1.03, 1.28)	<0.001	1.15 (1.04, ∞)	<0.001*
StO_2, %	1.10 (1.01, 1.24)	0.033	1.18 (1.01, 1.55)	0.040
ΔStO_2 from Baseline	1.23 (1.05, 1.56)	0.004	1.28 (1.06, 1.83)	0.004
rStO_2 to Baseline, %	1.07 (1.01, 1.16)	0.013	1.13 (1.02, 1.31)	0.010
ΔStO_2 from 1min-CPR	1.63 (1.17, 3.36)	<0.001	2.50 (1.21, ∞)	<0.001*
rStO_2 to 1min-CPR, %	1.14 (1.04, 1.33)	<0.001	1.28 (1.06, ∞)	<0.001*
THC, µmol/L	1.05 (0.93, 1.20)	0.453	1.06 (0.93, 1.22)	0.428
ΔTHC from Baseline	1.26 (1.06, 1.63)	0.002	1.23 (1.03, 1.65)	0.013
rTHC to Baseline, %	1.18 (1.05, 1.42)	0.002	1.16 (1.02, 1.41)	0.012
ΔTHC from 1min-CPR	1.34 (1.08, 2.01)	0.001	1.37 (1.05, 2.42)	0.006
rTHC to 1min-CPR, %	1.24 (1.06, 1.65)	0.002	1.23 (1.03, 1.70)	0.008

*Median unbiased estimate with one-sided p-value.

Definition of abbreviations: [HbO_2] = oxy-hemoglobin concentration; [Hb] = deoxy-hemoglobin concentration; StO_2 = tissue oxygen saturation; THC = total hemoglobin concentration; Δ-prefix = absolute change; r-prefix = relative value compared to 100% at baseline.
